# Premature Delivery for the Prevention of Blindness

**Published:** 1883-03-20

**Authors:** Edward G. Loring

**Affiliations:** New York


### PREMATURE DELIVERY FOR THE PREVENTION OF BLINDNESS.

By EDWARD G. LORING, M.D., NEW YORK.

So far as I know, and so far as I have been able to inform myself by inquiries among my professional brethren, premature delivery for the sole purpose of preventing blindness has never, up to the present time, been performed, or even advocated.

In suggesting any new remedy, or remedial procedure, especially in so conservative an art as medicine, two things ought to be considered: first, whether there is any necessity for the remedy proposed, and, secondly, whether the advantages attending its adoption will outweigh the evil effects which existed before the remedy was suggested.

First, as to the necessity of the operation.

It has been long known that pregnant women, especially toward the end of gestation, were liable to suffer from a disturbance of vision, which might vary from the slightest deterioration to a total and permanent blindness.

Physicians were well aware of this fact before the true condition of retinitis albuminuria, or uræmic amaurosis, was known—that is to say, prior to the time of Bright, and long before the invention of the ophthalmoscope.

The fact, therefore, admitted, that possible, and, under some conditions, inevitable blindness may ensue, the question reduces itself to the simple inquiry whether premature delivery is ever justifiable either for the restoration or the preservation of sight.

It appears to the writer that there are not a few cases in which it is not only justifiable, but where the true principles of sound practice demand its adoption.

The reasons for this belief will, perhaps, be better explained by the following cases, which are cited simply as useful examples of what may happen, and not unfrequently does happen, either directly or indirectly, in the experience of every physician and oculist.

The following case is reported by Mr. Robert Lee, the particulars being furnished to him by Mr. Bowman.

On the 7th of December Mr. Bowman was consulted by a lady who was pregnant, and "who had rather suddenly lost her sight in great measure." The urine was found to be excessively loaded with albumen. At Mr. Bowman's suggestion the patient consulted Mr. Paget and Dr. West, who anticipated, Mr. Bowman says, as he did himself, "that the confinement would be attended with convulsions of a dangerous character." On the 9th of January, or one month later, the patient had a premature confinement of a male fœtus, which was still-born. The patient gradually regained her health and strength, but Mr. Bowman regrets to state that the sight has only partially returned.

The first thing which strikes one here is that there was a permanent loss of vision, and the question arises whether something might not have been done to prevent it. It is stated that on the 7th of December the patient had rather suddenly lost her sight, and, if there ever was a chance of saving it, it was by getting rid of the cause as soon as possible, as it is universally acknowledged by all oculists that the sooner the delivery takes place the better the prognosis for sight. Nothing, however, was done, notwithstanding the fact that the three attending physicians, all men of great eminence, had announced that they anticipated that when the confinement did take place it would be attended with dangerous convulsions.

No action, however, was taken, and the woman would have been allowed to remain blind to the end of her term unless nature had stepped in with a premature birth. At any rate, she was allowed to remain blind for an entire month, with the urine loaded with albumen; and what a month's delay, under these circumstances, will, and often does produce in so delicate a tissue as that of the retina and optic nerve is too well known to need any comment. Would it not have been better practice—nay, was it not the bounden duty of the surgeons in a case in which a great degree of blindness was actually present, and danger to life anticipated, to have produced premature delivery, and not waited for an entire month for the chance intervention of nature, and to have, in the end, to regret that "the sight had only partially been restored"? Does not the mere fact that the sight did partially return after the patient had lost it for a month show that if the delivery had taken place earlier there would have been every chance of its being fully restored? and is the sight of a woman in the prime of life of so little account that no effort should be made, or no risk run, to preserve it, especially when it is anticipated that, later on, there will be danger to her life itself?

The literature for the last half century, as everybody is aware, is full of such cases, and that related above is cited simply for purposes of illustration.

The following case, by Mr. Lawson, is a sad example of the misery which may be entailed upon a patient by recurrent attacks of blindness occurring in successive pregnancies:

P. S., aged forty-one, applied to the hospital on December 2d of this year, suffering from the following amaurotic symptoms: She was the mother of nine children, six of whom were then living. She said that in the second month of her pregnancy with her *eighth* child her sight began to fail her, and continued to get worse until the termination of her pregnancy. She could then only see large objects; she could not count fingers, nor tell a man from a woman. She could merely see that something large was in front of her. After the birth of her child her sight began to improve, but not until after she had begun regularly to suckle it; and in three months time she was able to read her Bible, the print of which was about the size of No. 10 Jæger, and she could not only see to do needle-work, but actually did it. In this state she continued for two years, when she again became pregnant with her *ninth* child, and her sight, at about the second month, again began to fail her, and continued to diminish until, at the ninth month, she could see no more than she did at the time of her previous confinement. After the birth of this child her sight only slighly improved compared with the improvement which followed the birth of the previous one. This she attributes to the fact of her being unable to suckle her child on account of her bad health. She regained, however, sufficient vision to be able to walk about unguided and to carry her child with her in the streets; and, although she could easily distinguish the faces of her friends, still she had not sufficient sight to be able to read or write. Eighteen months have elapsed, and she is now pregnant with her *tenth* child. The increased impairment of vision came on about the second month as on the two previous occasions, but her sight has failed her this time mnch more rapidly, and on that account she applied to the hospital.

She is now six months advanced in pregnancy, and the following is the condition of her sight:

*Right Eye.*—No perception of light; pupil fixed, widely dilated.

*Left Eye.*—Is unable to count fingers, but at eight inches can just make out the hand; pupil dilated, but with a very slight range of action.

*Examined with the Ophthalmoscope.*—The optic entrance looks small, of a bluish pearly white; the arteries appear like mere threads, while the veins are very large.

She suffers now, and has suffered during her pregnancies, with pain at the top and back of her head. The least noise "seems to bewilder her," and she arises in the morning with headache. She has no loss of power in any of her limbs, but looks thin and haggard, having the appearance of one who has gone through much trouble.

A striking feature in this case, and one which is thought to be uncommon, though it is by no means so rare as is supposed, is that the vision began to fail so early in the pregnancy—that is, about the second month—and that it occurred in three successive pregnancies at this time. Now, notwithstanding this fact, and that with each pregnancy there was an additional and serious increase in the loss of the sight, the patient was allowed to go through three successive pregnancies until she became totally blind without an effort being made to prevent it. No wonder she looked, as the reporter of the case expresses it, "thin and haggard, having the appearance of one who has gone through much trouble."

To be blind at forty-one with eight or ten children to look after is not a cheerful prospect for any woman. To be allowed to become so through a succession of years would seem, if possible, only to add to the misery. Other cases precisely similar might be cited here, as they are familiar to every accoucheur.

Such being the condition of affairs, when matters are left to themselves, it remains to be seen what occurs when interference, either natural or artificial, takes place, and in illustrating this I would briefly cite another case from Mr. Lee :

On the 29th of January, 1883, Mr. Lee saw a young lady in the third month of her pregnancy. She was suffering at that time from sensitiveness in the region of the uterus, sickness of stomach, and general nervous irritability ; the pulse, however, was not very rapid, and there were no symptoms to excite alarm.

On March the 21st, or two months later, he received a note from Mr. Bowman desiring a consultation, as the patient had consulted him for defective vision. "The urine was loaded with albumen, and there was a destructive disease going on in the coats of the eye." As the patient was in the sixth month of her pregnancy, and as she seemed to be threatened with convulsions, and espe-pecially as these symptoms became urgent in the following three days, premature delivery was proposed by Mr. Lee, as he believed the affection of the kidneys and eyes arose from the pregnancy. Before, however, having recourse to this, it was considered proper to hold a consultation with Dr. Robert Ferguson, who thought it unadvisable to induce premature labor, "chiefly on the ground that the life of the child would necessarily be sacrificed, as the pregnancy had not advanced beyond the sixth month." It was determined, in consequence of this opinion, not to interfere, but to wait and see what course the disease would take.

On April 10th, or three weeks later, there was an attack of convulsions; and, as these were sufficient to excite apprehensions that fatal puerperal convulsions would take place, it was considered proper, after a second consultation, that premature labor should be induced immediately. This was successfully performed, and, with occasional drawbacks, the patient continued to recover for the next ten days, when she could distinctly see the figures on the dial of a watch.

The happy result in this case and the restoration to vision were, it cannot be denied, due to the induction of premature delivery, which removed the cause producing the loss of sight; and the only criticism to be made is that it was not induced as speedily as it should have been, since the operation was delayed until dangerous convulsions had already taken place, notwithstanding the fact that precisely this result had been anticipated, and in spite, also, of the fact that "a destructive disease was going on in the coats of the eye, and that the urine was at the same time loaded with albumen." Neither would it seem that the reason given for the delay —that the life of the child would be sacrificed—was a good and sufficient one, since, from the very nature of the trouble and the condition of the mother, there was every reason to suppose that, sooner or later, not only the life of the child was sure to be sacrificed, as it ultimately was, but also that of the mother would be imperiled. Here, too, the question narrows itself to whether the *possible* life of a sickly child ought to outweigh the probable loss of sight in the mother, and whether it is good practice, or even justifiable, to run such a risk.—*N. Y. Med. Journal.*

